# Emergency endovascular management of peripheral artery aneurysms and pseudoaneurysms – a review

**DOI:** 10.1186/1749-7922-3-22

**Published:** 2008-07-21

**Authors:** Umar Sadat, Peter J Kullar, Ayesha Noorani, Jonathan H Gillard, David G Cooper, Jonathan R Boyle

**Affiliations:** 1 Cambridge Vascular Unit, Cambridge University Hospitals NHS Foundation Trust, UK; 2 University Department of Radiology, Cambridge University Hospitals NHS Foundation Trust, UK

## Abstract

Endovascular stenting has been successfully employed in the management of aortic aneurysms; however, its use in managing peripheral arterial conditions remains questionable. We review the utility of endovascular technique in the management of peripheral arterial conditions like aneurysms, pseudoaneurysms and arterio-venous fistulas in the emergency setting. Though long term data about graft patency rates is not yet available, the endovascular approach appears to be a useful minimally invasive technique in situations where open repair is either difficult or not feasible.

## Background

Popliteal aneurysms are the most common peripheral artery aneurysms, followed by femoral artery aneurysms. Patients with popliteal artery aneurysms have a 70% risk of concurrent abdominal aortic aneurysm (AAA). Similarly, patients with AAA have a 3.1% risk of popliteal aneurysm. Patients have bilateral popliteal artery aneurysms in 50–70% of cases [1]. Patients with popliteal artery aneurysms usually present with critical limb ischemia, resulting from aneurysm thrombosis. Rupture of the popliteal aneurysms is uncommon.

Femoral aneurysms are asymptomatic in 30–40% of patients at the time of initial presentation. Approximately one third of patients presents with local symptoms such as groin pain or groin mass. Of patients presenting with femoral aneurysms 10–65% have complications at the time of initial presentation, including chronic thrombosis with claudication, acute thrombosis with limb ischaemia and rupture. Distal embolisation is less common. The majority of patients with femoral aneurysms have multiple aneurysms, with a 51–92% incidence of associated aorto-iliac aneurysmal disease [2]. Peripheral arterial aneurysms have a male predominance. Common risk factors include smoking, hypertension and atherosclerosis.

Historically, aneurysms have been repaired by open technique, but over the last two decades endovascular stenting has been increasingly used for the management of aneurysmal and stenotic arterial conditions, particularly in the aorta. Different randomized controlled trials and comparative studies have shown early reduction in postoperative morbidity and mortality [3]. But considerably longer follow up and re-intervention rates are some of the concerns related to the endovascular approach. Though this evidence stems from studies in the elective setting, no completed randomized controlled trials in emergency settings have been done to date. But, it would be unrealistic to assume that the limitations of endovascular stenting in the elective setting should limit its utility in the emergency setting. This is because this minimally invasive technique has significant benefits in the acute settings most importantly less surgical stress [3]. This can be useful in patients who have significant co-morbidities and are otherwise not fit for open surgery [4]. There is much less evidence about the usefulness of endovascular stenting in peripheral arteries.

## Management

### Peripheral aneurysmal disease

**Figure 1:**
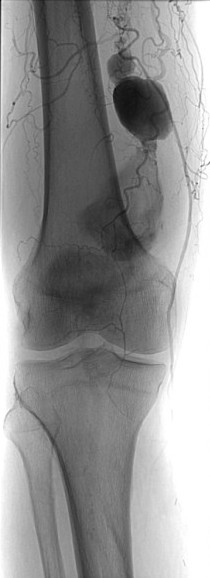
Popliteal artery aneurysm.

**Figure 2:**
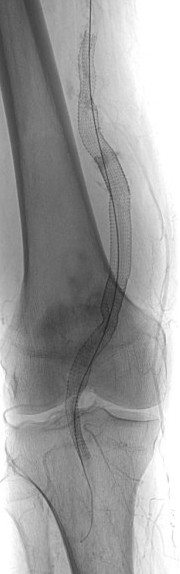
Complete exclusion of popliteal aneurysm following endovascular stenting.

The first report of popliteal aneurysm stenting was by Marin et al when a Palmaz stent covered with polytetrafluoroethylene (PTFE) was used [5]. Since then there have been reports of its use in treating popliteal aneurysms, with results equivalent to that of open technique. In a recent meta analysis, it was reported that endovascular technique offers similar medium term benefits as an open repair [6]. There is only one randomised controlled trial that has reported comparable long-term results for endovascular repair compared with open repair [7]. The position of the endograft behind the knee also makes the situation more complex. The flexion at the knee joint may possibly lead to kinking of the stent, predisposing to stent blockage/fracture [8,9]. But careful endograft design and placement can help overcome this problem to an extent (Figure 1, 2). There are similar concerns about patients with femoral aneurysms who undergo endovascular stenting because of the movement at the hip joint. We have already reported the successful use of this technique in managing popliteal artery aneurysm in "hostile leg" [10]. Further studies are warranted before it can be adopted as a treatment of first choice.

### Arterio-venous fistulas and pseudoaneurysms

Endovascular stenting can also be used in treating arterio-venous fistulas/malformations and pseudoaneurysms in elective and emergency setting. This can be done by using covered stents or by coil embolization. There are many case reports in which it has been used to treat femoral [11,12], popliteal [13,14], tibial pseudoaneurysms [15,16] and arterio-venous malformations [17,18]. In our experience this is a safe, minimally-invasive intervention which ensures an early return to work and a minimal hospital stay [19]. The importance of endovascular stenting is quite significant in patients who develop pseudoaneurysms following orthopaedic procedures like total or partial knee replacement or during knee arthroplasty. These can present in both emergency and elective settings. Because of the hostile operative field, the endovascular approach offers a quick fix to the problem. Both covered stents and coils can be used depending upon the nature and extent of the arterial injury.

**Figure 3:**
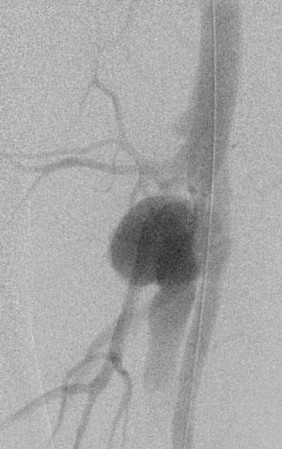
Arterio-venous fistula of femoral artery.

**Figure 4:**
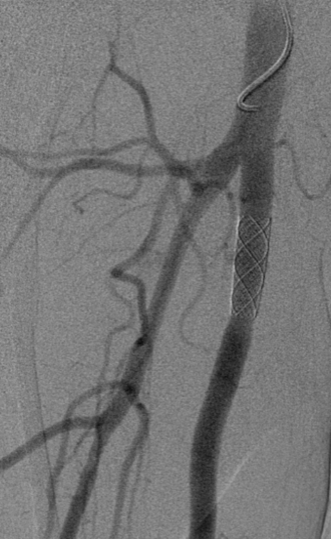
Complete exclusion of arterio-venous fistula following endovascular repair.

To avoid working in a difficult hostile operative field, this method is quite useful in managing patients with infectious arterio-venous fistulas/malformations. These are particularly common in intravenous drug abusers. We have already reported a case in which it was used to treat arterio-venous fistulas with a concurrent femoral pseudoaneurysm in an intra venous drug abuser, where open surgery was not possible due to the presence of a groin abscess (Figure 3, 4). Appropriate intravenous antibiotic cover is important in this scenario, to prevent graft infection [20].

## Conclusion

The endovascular approach being minimally invasive, offers considerable benefits in the emergency setting, though long-term data about its efficacy is awaited.

## Conflict of interests

The authors declare that they have no competing interests.
